# Analysis of Digoxin and Metildigoxin in Whole Blood Using Solid-Phase Extraction and Liquid Chromatography Tandem Mass Spectrometry

**DOI:** 10.1155/2012/975824

**Published:** 2011-06-22

**Authors:** Paula Melo, Rita Machado, Helena M. Teixeira

**Affiliations:** ^1^National Institute of Legal Medicine, North Branch, 4050-167 Porto, Portugal; ^2^Forensic Sciences Centre (CENCIFOR), 3000-213 Coimbra, Portugal; ^3^Faculty of Sciences and Technology, University of Coimbra, 3030-790 Coimbra, Portugal; ^4^Faculty of Medicine, University of Porto, 4099-002 Porto, Portugal; ^5^Faculty of Medicine, University of Coimbra, 3000-548 Coimbra, Portugal

## Abstract

A simple and rapid UPLC/MS/MS method has been developed and validated for the analysis of digoxin and metildigoxin in whole blood. Samples were prepared by SPE extraction with Oasis HLB columns. Separation was achieved with an ACQUITY UPLC HSS T3 column (2.1 × 100; 1.8 *μ*m), at 35°C. The mobile phase consisted of acetonitrile (70%) and ammonium formate 5 mM (30%). Total run time was 1.5 min operating at isocratic mode with a flow rate of 0.3 mL/min. Mass spectrometry detection was performed by positive mode *electrospray*, on the ammonium adducts with two transitions for each analyte and one for the IS (d_3_-digoxin). The method proved to be specific and linear over the range (0.3–10) ng/mL. This technique also showed high sensitivity with a 0.09 ng/mL LOD and a 0.28 ng/mL LOQ, for both substances. Percentage recovery ranged from 83 to 100% for digoxin and from 62 to 94% for metildigoxin. The intra- and interday precision CV were ≤10%.

## 1. Introduction

**Figure 1:**
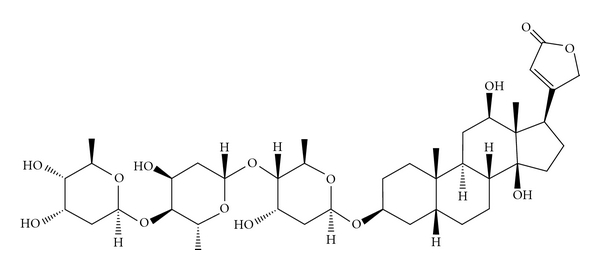
Chemical structure of digoxin (C_41_H_64_O_14_).

**Figure 2:**
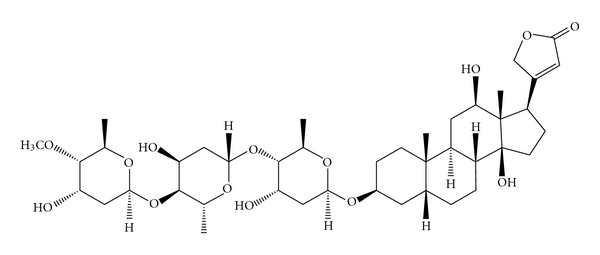
Chemical structure of metildigoxin (C_42_H_66_O_14_).

Digitalis glycosides are the drugs of choice for the treatment of congestive heart failure and certain disturbances in cardiac rhythm, producing a positive inotropic activity and increase, myocardial contractility [1]. Some authors state that digoxin is clinically the most commonly used cardiac glycoside [2]. Nevertheless, in Portugal, metildigoxin is also a common cardiac glycoside, being the second more frequently used [3]. Digoxin (Figure 1) is metabolised mainly in the liver [2], by stepwise removal of the sugar moieties to form digoxigenin, which is further metabolised to inactive metabolites which may be excreted in free or conjugated forms. Reduction to dihydrodigoxin, relatively inactive, also occurs [4]. However, digoxin is metabolised in man in a small percentage [5]. Effectively, digoxin is excreted mainly unaltered in the urine, with up to 80% of a dose excreted in urine in 7 days being 27% of the dose excreted in the first 24 h [4]. Digoxin can be found as the result of its administration or due to metabolism since it is a metabolite of deslanoside, digitoxin, lanatoside C and metildigoxin [4]. Metildigoxin (Figure 2) is metabolized by demethylation into digoxin and by hydrolysis into the bis- and monoglycosides. About 75% of a dose is excreted in urine over a several days period, with about 25–30% excreted in the first 24 h. About 30–50% of the material excreted in urine corresponds to unchanged drug, being 15% conjugated bis- and monoglycosides and the remains are digoxin [4]. 

These compounds have a narrow therapeutic range, so they can frequently lead to intoxication, involving suicide, homicide, and accidental poisoning cases [2]. When this intoxication is lethal, legal procedures are implicated, and the forensic laboratories must confirm the existence of cardiac glycosides in the collected samples to help determine if digitalis intoxication was, in fact, the cause of death. Therefore, it is essential that toxicology laboratories are capable to detect and quantify this group of substances in *postmortem* samples.

In this study, we have developed a sensitive and rapid method for the identification and quantification of digoxin and metildigoxin in whole blood by UPLC-MS/MS, after a SPE extraction technique.

## 2. Experimental

### 2.1. Instrumentation

Chromatographic separation was carried out on an ACQUITY UPLC system (Waters) with an ACQUITY TQD Mass Detector (Waters). Solid-phase extractions were carried out on an automatic extractor Aspec XL (Gilson).

### 2.2. Materials, Standards, and Chemicals

Pure digoxin, metildigoxin, and d_3_-digoxin (internal standard) were purchased from Chemos (Germany). Each standard compound was dissolved in methanol (1 mg/mL for digoxin and 0.5 mg/mL for metildigoxin and d_3_-digoxin) and stored at −20°C. Working solutions were also prepared in methanol. Ammonium formate was purchased from Sigma-Aldrich (USA). Ammonium acetate was purchased from Merck (Darmstadt, Germany). All the other solvents were analytical or HPLC grade and were purchased from E. Merck (Darmstadt, Germany). Water was purified by a Milli-Q system obtained from Millipore (Molsheim, France). The mobile phase was filtered with a 0.20 *μ*m Schleicher & Schuell filter and degassed in an ultrasonic bath for 15 minutes just before use. Oasis HLB (3 cc; 60 mg) solid-phase extraction columns were obtained from Waters (Via Athena, Portugal).

### 2.3. UPLC-MS/MS Conditions

Chromatographic separation was performed with an ACQUITY UPLC HSS T3 column (2.1 × 100; 1.8 *μ*m particle size), at 35°C. The mobile phase consisted of acetonitrile (70%) and ammonium formate 5 mM (30%). Total run time was 1.5 min operating at isocratic mode with a flow rate of 0.3 mL/min. The injection volume was 10 *μ*L (full loop), and the sample manager temperature was 10°C. Mass spectrometry detection was carried out using electrospray ionization operating at positive mode. The main other instrumental settings were capillary voltage 2 KV; cone voltage 30 V; extractor 1 V; ion energy_1_ 1; Ion energy_2_ 3; source temperature 120°C; desolvation temperature 300°C; cone gas flow rate 0 L/h, desolvation gas flow rate 500 L/h. Multiple reaction monitoring (*MRM*) was used to detect digoxin and metildigoxin, with two transitions of ammonium adduct of each analyte, one for quantitation and the other for confirmation. The appropriate MRM transitions, cone voltages and collisions energies are described in Table 1. d_3_-digoxin was used as internal standard. Instrument control, data acquisition, and process were achieved by the use of Waters MassLynx software (Milford, Mass).

### 2.4. Sample Preparation and SPE Extraction

Control and calibration samples were prepared by spiking drug-free *postmortem* blood samples with standard solutions. One milliliter of drug-free whole blood samples was first spiked with the substances at concentrations ranging from 0.3 to 10 ng/mL. Deuterated internal standard (IS), d_3_-Digoxin, was used by adding 50 *μ*L of a 100 ng/mL solution to all the samples examined. Five hundred microliters of ammonium acetate 2 M solution (pH = 9.5) and 3.5 mL Mili-Q water were added and flowed by agitation on a vortex mixer. The samples were then centrifuged for 15 minutes at 3200 rpm before extraction, and the supernatant decanted to another tube. A solid-phase extraction (SPE) technique was carried out to isolate the analytes, using Oasis HLB columns (3 cc; 60 mg). SPE columns were conditioned by sequentially adding 1 mL of methanol, 1 mL of water, and 3 mL of a 0.1 M ammonium acetate solution (pH = 9.5). The prepared samples were poured onto the conditioned columns and allowed to drain. Each column was then washed with 2 mL of a 0.1 M ammonium acetate solution (pH = 9.5) and dried under maximum vacuum for 2 min. The cardiac glycosides were then eluted with 3 mL of chloroform:2-propanol (95 : 5). The solvent was evaporated to dryness at 25°C under gentle nitrogen flow. Residues were redissolved with 100 *μ*L of acetonitrile/water (60 : 40) and vortexed to increase recovery from tube walls. Ten microlitres of the final solution was then injected into the UPLC-MS/MS system.

## 3. Results and Discussion

### 3.1. Method Development

There are various studies in the literature reporting the development of methods for digoxin determination in human plasma, serum, and urine by liquid chromatography-mass spectrometry LC-MS [6–12], and some describe the determination of digoxin in rat urine or plasma [13, 14]. However, only few data have been published about digoxin analysis in whole blood samples [15–17]. Furthermore, to the best of our knowledge, the present study is the first one to report a validated method for the analysis of metildigoxin, the second more frequently used cardiac glycoside in Portugal, in whole human blood. On the other hand, only one publication reports the analysis of digoxin by UPLC, but the study was performed in rat urine [13]. Thus, it is important to develop new methods concerning the analysis of these digitalis glycosides in whole blood samples, the most frequently analysed matrix in *postmortem* cases. 

**Figure 3:**
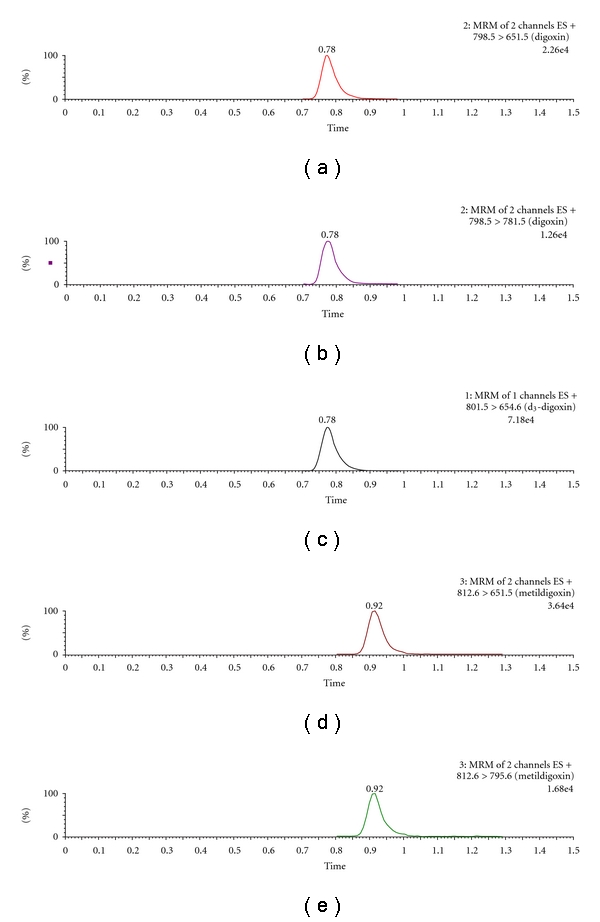
MRM chromatograms of whole blood samples spiked with 1 ng/mL of digoxin [(a) and (b)] and metildigoxin [(d) and (e)]; and with 5 ng/mL of d_3_-digoxin, internal standard (c).

In the present study, a sensitive and rapid UPLC-MS/MS method for the determination of digoxin and metildigoxin in *postmortem* blood samples was developed and validated, with sufficient chromatographic separation (Figure 3) and precise quantification of the studied substances. Moreover, the use of this new UPLC technique with tandem mass spectrometry makes the developed method a simple and suitable technique for routine forensic analysis, with less time-consuming analysis and more sensitivity and specificity. 

**Figure 4:**
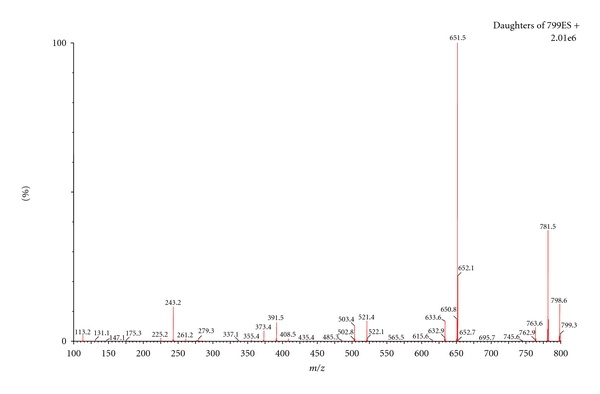
Product ion mass spectra of digoxin.

**Figure 5:**
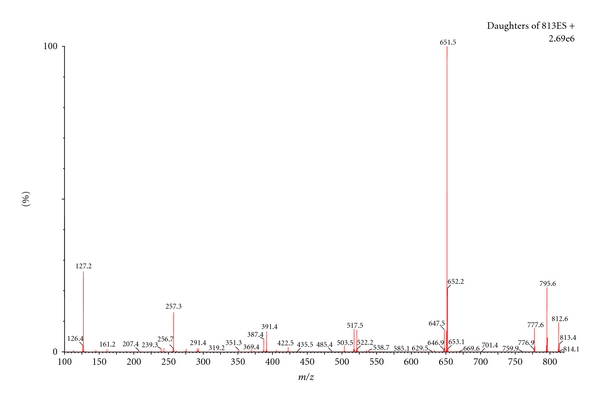
Product ion mass spectra of metildigoxin.

Thus, validation studies were performed separately for each substance, in whole blood samples. The chromatographic method described had a run time of only 1.5 min, with digoxin detection at a retention time of 0.78 and 0.92 min for metildigoxin (Figure 3). The ionization of the studied cardiac glycosides by *electrospray* ionization (ESI) in positive mode achieved the best results, since the positive ion full-scan mass spectra of both compounds demonstrated that the ammonium adduct ion in the positive mode showed very strong intensity compared to the [M-H]^−^ in the negative mode. Consequently, ammonium adduct of both compounds was selected as the precursor ion. The product ions were obtained by the ammonium adduct precursor fragmentation in a collision cell. The compounds were quantified employing the *MRM* mode using the transitions 798.5 > 651.5 for digoxin and 812.6 > 651.5 for metildigoxin, and the transitions for confirmation, 798.5 > 781.5 for digoxin, and 812.6 > 795.6 for metildigoxin (Table 1). Figures 4 and 5 represent the product ion mass spectra of digoxin and metildigoxin, respectively.

### 3.2. Validation of the UPLC-MS/MS Method

The developed method was validated according to the guidelines of the International Conference on Harmonization (ICH) for the validation of the following parameters: selectivity/specificity, linearity, limits of detection and quantification, recovery, and intra- and interday precision [18].

#### 3.2.1. Specificity

To evaluate peak purity and selectivity, 10 different blank samples (no analyte or internal standard was added) were analyzed to check for peaks that might interfere with the detection of the analyte or internal standard (IS). Also, negative samples (blank blood samples + IS) were analyzed, to verify the absence of native analyte in the IS solution. In addition, ten blood samples spiked with the 2 analytes but without IS were analysed, in order to verify no interferences from the analytes in the IS retention time. To assess possible interferences, 10 different samples were spiked with a mixture of various benzodiazepines (bromazepam, nordiazepam, 7-aminoflunitrazepam, flunitrazepam, oxazepam, diazepam, clonazepam, temazepam and midazolam) at a concentration of 25 ng/mL and with digoxin and metildigoxin at 5 ng/mL. All 10 samples were free of coeluting peaks at the retention times of the corresponding studied substances and their respective deuterated IS. Analysis of negative samples also demonstrated that the IS did not contain relevant amounts of native cardiac glycosides. None of the 10 compounds tested showed any interference when added to a blood sample with 5 ng/mL digoxin and metildigoxin.

#### 3.2.2. Limit of Detection (LOD) and Quantitation (LOQ)

The limit of detection (LOD) was estimated from extracted samples spiked with decreasing concentrations of the studied compounds. The limits of detection (*LOD*) and quantitation (*LOQ*) were established using the standard deviation of the response (*σ*) and the slope of the linear regression (S), as *LOD* = 3.3 *σ*/S and *LOQ *= 10 *σ*/S. This technique also showed high sensitivity with a 0.09 ng/mL LOD and a 0.28 ng/mL LOQ, for both substances, digoxin and metildigoxin.

#### 3.2.3. Linearity

Simple linear regression analyses were performed, with calibration curves constructed from peak area ratios, spiking whole blood samples with the studied substances at 10 different concentrations of the cardiac glycosides covering the range of (0.3–10) ng/mL. Ten calibrators were used to generate the standard curve, each calibrator injected in three aliquots. Calibration curves showed a linear relationship for digoxin with a correlation coefficient of 0.994 and for metildigoxin with a correlation coefficient of 0.996.

#### 3.2.4. Precision

Intraday and interday coefficients of variation values were determined by replicate analyses (*n* = 5) of *postmortem* spiked blood aliquots, either on the same run (intraday) or on five separate days (interday). Three concentration levels were selected for validation (1, 3, and 8 ng/mL). The data on precision are presented in Tables 2 and 3. For digoxin and metildigoxin, good precision values were obtained, shown by the low percent values clearly below 15%, at the three concentration levels studied.

#### 3.2.5. Extraction Recovery

The recovery of SPE was determined by repeated analysis of five samples spiked at three different levels of digoxin and metildigoxin concentration (1, 3, and 8 ng/mL). The extraction recovery was determined by comparing the representative peak areas of extracted drug-free samples spiked before extraction with the peak area of drug-free samples fortified after the extraction at the same concentration levels. Tables 2 and 3 also show that the calculated extraction efficiencies for digoxin ranged from 83 to 100% and from 62 to 94%, for metildigoxin. The method provided good extraction efficiencies for digoxin at all concentration levels. However, for metildigoxin, lower (but still good) recoveries were achieved, especially in lower concentrations. No significant matrix effect was observed for the analytes in the analyzed samples. Sample preparation is extremely important to the overall method with respect to increasing the sensitivity and reducing possible interference from the sample matrix. The extraction technique employed allowed good recovery and appropriate selectivity and was, at the same time, simple and reproducible.

## 4. Conclusion

In summary, this paper describes an UPLC-MS/MS procedure for quantitative analysis of digoxin and metildigoxin in *postmortem* whole blood samples. The procedure presented here has high specificity, selectivity, and sensitivity and very good limits of detection and quantification and can be regarded as an alternative method to detect and quantify therapeutic levels of these cardiac glycosides [5, 19], being less demanding and time consuming. In fact, this methodology proved to be less laborious, overcoming some disadvantages of the existing methodologies. The method also proved to be selective and sensitive for a reduced sample volume, despite the very low therapeutic levels, which is a distinct advantage for the accomplishment of toxicological analysis in forensic toxicology.

## Figures and Tables

**Table 1:** *MRM* transitions, cone voltage and collision energy for digoxin, metildigoxin, and d_3_-digoxin (IS).

Compound	Transition	Cone voltage (V)	Collision energy
Digoxin	798.5 > 651.5	30	16
	798.5 > 781.5	30	8
Metildigoxin	812.6 > 651.5	30	15
	812.6 > 795.6	30	9
d_3_-digoxin	801.5 > 654.6	30	15

**Table 2:** Recovery and precision for digoxin in spiked whole blood samples.

Concentration (ng/mL)	Mean (ng/mL)	Extraction recovery (%)	CV (%)	
			Intraday	Interday
1	1.11 ± 0.13	88 ± 7	4.56–10.00	7.80
3	3.27 ± 0.23	83 ± 2	1.95–7.35	4.83
8	7.42 ± 0.70	100 ± 3	0.78–9.91	5.82

**Table 3:** Recovery and precision for metildigoxin in spiked whole blood samples.

Concentration (ng/mL)	Mean (ng/mL)	Extraction recovery (%)	CV (%)	
			Intraday	Interday
1	1.21 ± 0.08	62 ± 5	4.56–9.90	7.45
3	3.23 ± 0.26	67 ± 3	4.82–9.62	7.51
8	7.96 ± 0.78	94 ± 8	2.32–9.80	6.65
